# IL-6 in the Cerebrospinal Fluid Signals Disease Activity in Multiple Sclerosis

**DOI:** 10.3389/fncel.2020.00120

**Published:** 2020-06-23

**Authors:** Mario Stampanoni Bassi, Ennio Iezzi, Jelena Drulovic, Tatjana Pekmezovic, Luana Gilio, Roberto Furlan, Annamaria Finardi, Girolama Alessandra Marfia, Francesco Sica, Diego Centonze, Fabio Buttari

**Affiliations:** ^1^Unit of Neurology and Neurorehabilitation, IRCCS Neuromed, Pozzilli, Italy; ^2^Clinic of Neurology, Clinical Center of Serbia, Belgrade, Serbia; ^3^Faculty of Medicine, University of Belgrade, Belgrade, Serbia; ^4^Faculty of Medicine, Institute of Epidemiology, University of Belgrade, Belgrade, Serbia; ^5^Clinical Neuroimmunology Unit, Institute of Experimental Neurology, Division of Neuroscience, San Raffaele Scientific Institute, Milan, Italy; ^6^Multiple Sclerosis Clinical and Research Unit, Department of Systems Medicine, University of Rome “Tor Vergata”, Rome, Italy; ^7^Synaptic Immunopathology Lab, Department of Systems Medicine, Tor Vergata University, Rome, Italy

**Keywords:** interleukin-6, multiple sclerosis, cerebrospinal fluid, MS diagnosis, MS prognosis, cytokines, progressive, relapsing–remitting

## Abstract

Specific proinflammatory and anti-inflammatory molecules could represent useful cerebrospinal fluid (CSF) biomarkers to predict the clinical course of multiple sclerosis (MS). The proinflammatory molecule interleukin (IL)-6 has been investigated in the pathophysiology of MS and has been associated in previous smaller studies to increased disability and disease activity. Here, we wanted to further address IL-6 as a possible CSF biomarker of MS by investigating its detectability in a large cohort of 534 MS patients and in 103 individuals with other non-inflammatory neurological diseases. In these newly diagnosed patients, we also explored correlations between IL-6 detectability, MS phenotypes, and disease characteristics. We found that IL-6 was more frequently detectable in the CSF of MS patients compared with their control counterparts as significant differences emerged between patients with Clinically isolated syndrome (CIS), Relapsing–remitting (RR), and secondary progressive and primary progressive MS compared to non-inflammatory controls. IL-6 was equally present in the CSF of all MS phenotypes. In RR MS patients, IL-6 detectability was found to signal clinically and/or radiologically defined disease activity, among all other clinical characteristics. Our results add further evidence that CSF proinflammatory cytokines could be useful for the identification of those MS patients who are prone to increased disease activity. In particular, IL-6 could represent an interesting prognostic biomarker of MS, as also demonstrated in other diseases where CSF IL-6 was found to identify patients with worse disease severity.

## Introduction

Multiple sclerosis (MS) is an autoimmune inflammatory disease of the central nervous system (CNS) characterized by a highly variable clinical course. Relapsing–remitting (RR)-MS is characterized by periods of clinical stability and acute relapses. Conversely, in progressive phenotypes (i.e., secondary progressive, SP-MS, and primary progressive, PP-MS), clinical decline can be observed without new acute inflammatory events.

Exacerbated CNS inflammation has been associated with worse disease course both in animal models (i.e., experimental autoimmune encephalomyelitis) and in MS patients (Zeis et al., 2008; Centonze et al., 2009; Rossi et al., 2014). Indeed soluble mediators of inflammation released by activated lymphocytes and resident immune cells represent key regulators of the auto-immune response in MS. Specific proinflammatory molecules, including interleukin (IL)-1β, IL-6, IL-8, tumor necrosis factor (TNF), and interferon-gamma, promote immune cell infiltration through the blood–brain barrier, leading to demyelination and axonal damage (Göbel et al., 2018). In addition, experimental studies showed that proinflammatory molecules also alter neuronal functioning by promoting excitotoxic neurodegeneration. These observations have highlighted that neuroinflammation may represent an independent and an important cause of neurodegeneration since the early phases of MS. In contrast, anti-inflammatory molecules could reduce the immune damage of the CNS, modulating the immune response and thus exerting a beneficial effect on MS course, also promoting neuroprotection (Cheng and Mattson, 1995; Lobo-Silva et al., 2016).

Specific proinflammatory and anti-inflammatory molecules could represent useful cerebrospinal fluid (CSF) biomarkers to predict the clinical course of MS patients. In this regard, elevated CSF levels of various proinflammatory molecules have been associated with enhanced long-term disease activity and disability progression (Rossi et al., 2015; Stampanoni Bassi et al., 2018). In addition, one study has reported that detectable CSF IL-1β at the time of diagnosis in remitting MS patients influences midterm disease progression (Rossi et al., 2014), demonstrating, at the same time, a remarkable role of silent inflammation in MS course pathophysiology.

Among the crucial proinflammatory molecules, IL-6 has been particularly investigated in the pathogenesis of MS (Göbel et al., 2018). Thus, it has been previously reported that this molecule could negatively affect the disease course of MS, promoting the development of a higher level of disability and enhanced disease activity (Stelmasiak et al., 2000; Kimura et al., 2017; Stampanoni Bassi et al., 2018).

The aim of this study was to further investigate, in a large cohort of MS patients and control subjects, the clinical significance of IL-6 detectability in the CSF. Furthermore, we examined the potential role of CSF IL-6 detectability in predicting disease activity and the development of disability in MS.

## Materials and Methods

### MS Patients

A group of 543 consecutive MS patients admitted to the neurological clinics of University Tor Vergata Hospital and Neuromed Institute were enrolled in the study. As controls, 103 CSF samples were collected from patients affected by other non-inflammatory neurological diseases. The study was approved by the ethics committees of the University Tor Vergata Hospital in Rome and of the Neuromed Research Institute in Pozzilli, Italy, according to the Declaration of Helsinki. All patients gave written informed consent to the study.

MS diagnosis was made according to clinical, laboratory, and magnetic resonance imaging (MRI) parameters (Thompson et al., 2018). Radiologically isolated syndrome (RIS) was defined as the presence of MRI alterations suggestive of MS in patients without clinical symptoms. Clinically isolated syndrome (CIS) consisted of patients with a single clinical episode suggestive of MS, without evidence of dissemination in space and time. When dissemination in space and time was demonstrated in patients with a clinical course characterized by periods of clinical stability and acute relapses, the patients were classified as RR-MS. In patients presenting a progressive course after disease onset, diagnosis of PP-MS was made. Finally, SP-MS was defined in patients presenting an initial RR course followed by a progressive course.

The control group comprised 103 patients with non-inflammatory CNS and peripheral nervous system disorders, such as vascular leukoencephalopathy, metabolic and hereditary polyneuropathies, normal pressure hydrocephalus, functional neurological disorder, and spondylotic myelopathy.

In all cases, clinical evaluation, CSF collection, and brain and spine MRI were performed during hospitalization for diagnostic purposes. No immunoactive drugs were administrated before hospitalization. When needed, corticosteroids and/or immunoactive therapies were initiated only after lumbar puncture (LP).

The patients were examined by brain and/or spinal 1.5- or 3.0-T MRI, including dual-echo proton density, fluid-attenuated inversion recovery, T1-weighted spin-echo (SE), T2-weighted fast SE, and contrast-enhanced T1-weighted SE after intravenous gadolinium (Gd) infusion (0.2 ml/kg). A Gd-enhancing (Gd+) lesion was defined as an area of hyperintense signaling on contrast-enhanced T1-weighted images. An active MRI was defined as one showing new or enlarging T2-weighted lesions and/or contrast-enhanced T1-weighted lesions.

CSF was collected by LP performed in lateral decubitus. Immediately after LP, CSF was centrifuged (1,300 rpm, 10 min) to remove cellular elements and stored at −80°C until being analyzed using a Bio-Plex multiplex cytokine assay (Bio-Rad Laboratories, Hercules, CA, USA) according to the manufacturer’s instructions. The CSF concentrations of IL-6 were calculated according to a standard curve generated for the specific target and expressed in picograms per milliliter (pg/ml). When the cytokine concentrations were below the detection threshold, they were assumed to be 0 pg/ml.

Several clinical parameters were collected at the time of diagnosis during hospitalization. Disease duration was calculated as the interval elapsing between disease onset and the time of diagnosis. Disease onset was defined as the first episode of focal neurological dysfunction suggestive of MS. Clinical disability was assessed using the Expanded Disability Status Scale (EDSS; Kurtzke, 1983). Progression index was calculated as the EDSS at the time of diagnosis divided by disease duration. For each patient, the number of clinical relapses which occurred before LP was recorded. Disease activity at the time of LP was defined as the presence of either a clinical relapse and/or active lesions at the MRI.

### Statistical Analysis

To determine the significance of the different proportions, a chi-square test was used, and the mean values were compared by using ANOVA if the data distribution was normal. In case of non-normal distribution, non-parametric tests were used for testing the statistical significance. The predictive value of the investigated variables was assessed by logistic regression analysis since the dependent variable was dichotomous (disease activity: yes/no). All variables with significance levels of 0.05 by univariate analysis were included in the multivariate models. Two-tailed *p*-values less than 0.05 were considered as significant. Data were analyzed using the Statistical Package for the Social Sciences software (Advanced Statistics, version 17.0, Chicago, IL, USA).

## Results

### IL-6 Detectability in MS

The demographic and clinical characteristics of patients with MS and of controls, in whom CSF IL-6 detectability was analyzed, are presented in Tables 1, 2. CSF IL-6 was statistically significantly more frequently detected in MS patients in comparison to the controls (*p* = 0.001).

**Table 1:** Demographic, clinical, and paraclinical characteristics of multiple sclerosis patients and control subjects at the time of lumbar puncture.

Variables	MS patients	Controls	*p*
**Number**	543	103	
**Gender**			
Male	185 (34.1%)	43 (41.3%)	0.155
Female	358 (65.9%)	61 (58.7%)	
Age (years; mean ± SD)	37.9 ± 12.6	49.2 ± 13.4	**0.001**
**IL-6**			
Detected	449 (87.2%)	71 (68.9%)	**0.001**
Not detected	66 (12.8%)	32 (31.1%)	
**Disease duration** (years; mean ± SD)	3.0 ± 5.4		
**EDSS score** (median, IR)	2.0 (1.5)		
**Progression index** (mean ± SD)	15.6 ± 42.4		
**Disease course**			
RIS	28 (5.4%)		
CIS	98 (19.0%)		
RR-MS	329 (63.9%)		
SP-MS	21 (4.1%)		
PP-MS	39 (7.6%)		
**Number of relapses prior to LP**	1.3 ± 0.8		
(mean ± SD)
**Unique CSF OCB**			
Yes	429 (86.8%)		
No	65 (13.2%)		
**Disease activity**			
Yes	252 (57.4%)		
No	187 (42.6%)		

*Values in bold denote statistical significance. CIS, clinically isolated syndrome; CSF, cerebrospinal fluid; EDSS, expanded disability status scale; IL, interleukin; IR, interquartile range; LP, lumbar puncture; MS, multiple sclerosis; OCB, oligoclonal bands; PP-MS, primary progressive MS; RIS, radiologically isolated syndrome; RR-MS, relapsing–remitting MS; SP-MS, secondary progressive MS*.

**Table 2:** Demographic and clinical characteristics of patients with various multiple sclerosis phenotypes.

Variables	MS phenotypes			
	CIS	RR-MS	SP-MS	PP-MS
Number	98	352	21	39
Gender (M/F)	32/66	113/239	9/12	19/20
Age (years)	36.82 ± 11.39	35.99 ± 12.04	51.78 ± 10.97	49.54 ± 11.56
Disease duration (years)	1.20 ± 2.98	3.40 ± 5.91	6.34 ± 6.62	4.12 ± 5.16
EDSS (median, IR)	1.5 (1.0)	2.0 (1.5)	4.0 (3.0)	3.5 (5.5)
Progression index	19.17 ± 37.74	14.83 ± 43.67	8.06 ± 27.63	6.82 ± 27.41
OCB (detected/not detected)	64/34	249/73	17/4	36/4
Number of relapses prior to LP	1.09 ± 0.29	1.53 ± 0.83	0.90 ± 1.12	/
Disease activity at the time of LP (yes/no)	49/39	195/115	8/10	8/14

*CIS, clinically isolated syndrome; EDSS, expanded disability status scale; IR, interquartile range; LP, lumbar puncture; MS, multiple sclerosis; OCB, oligoclonal bands; PP-MS, primary progressive MS; RR-MS, relapsing–remitting MS; SP-MS, secondary progressive MS*.

### IL-6 Detectability and MS Phenotypes

The analysis of the distribution of patients with various MS phenotypes and of control subjects, according to CSF IL-6 detectability, demonstrated that IL-6 was more frequently present in CIS patients in comparison to controls [90/98 (91.8%) vs. 71/103 (68.9%), *p* = 0.001], RR-MS patients vs. controls [281/329 (85.4%) vs. 71/103 (68.9%), *p* = 0.001], SP-MS patients vs. controls [20/21 (95.2%) vs. 71/103 (69.7%), *p* = 0.013], and PP-MS [34/39 (87.2%) vs. 71/103 (68.9%), *p* = 0.027], respectively. There was no significant difference in CSF IL-6 detectability between patients with RIS and the controls [24/28 (85.7%) vs. 71/103 (68.9%), *p* = 0.078]. Additionally, a difference in CSF IL-6 detectability was not detected when comparing patients with relapsing (CIS and RR) and with progressive (SP and PP) disease (*p* = 0.130 and *p* = 0.293, respectively).

### IL-6 Detectability and MS Clinical Characteristics

Results of the correlation analyses between the CSF IL-6 detectability and the selected demographic, clinical, and paraclinical variables in the subgroup comprising RR-MS patients are presented in Table 3. In this subgroup of MS patients, a significant positive correlation was demonstrated between CSF IL-6 detectability and disease activity (*σ* = 0.115, *p* = 0.039). A significant correlation with CSF IL-6 detectability was not demonstrated with any other tested variable in this cohort of MS patients. Additionally, there was no correlation between IL-6 detectability and any of the tested variables in the remaining MS groups (RIS, CIS, SP-MS, and PP-MS).

**Table 3:** Correlation analysis between cerebrospinal fluid IL-6 detectability and selected demographic, clinical, and paraclinical variables in relapsing–remitting multiple sclerosis patients.

Variables	Correlation coefficient (*σ*)	*P*
Gender	0.051	0.361
Age (years)	−0.046	0.404
Disease duration (years)	−0.015	0.793
EDSS score	−0.078	0.158
Progression index	−0.033	0.555
Number of relapses prior to LP	−0.100	0.071
Unique CSF OCB	0.061	0.282
Disease activity	0.115	**0.039**

*Value in bold denotes statistical significance. CSF, cerebrospinal fluid; EDSS, expanded disability status scale; LP, lumbar puncture; MS, multiple sclerosis; OCB, oligoclonal bands; RR-MS, relapsing–remitting MS*.

**Figure 1:**
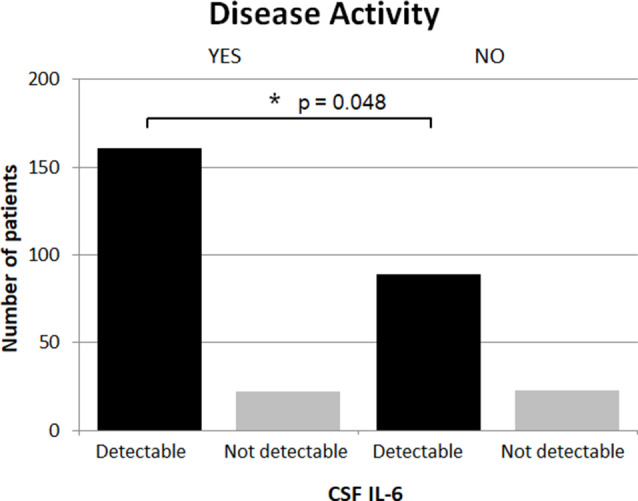
IL-6 detectability and disease activity in RR-MS patients. CSF, cerebrospinal fluid; IL, interleukin; RR-MS, relapsing–remitting multiple sclerosis.

Having in mind the demonstrated positive correlation between CSF IL-6 detectability and disease activity in our RR-MS patients, we compared the distribution of detectable CSF IL-6 between active RR-MS patients and controls. It has been shown that, in the active MS patients from the abovementioned group, IL-6 detectability was significantly higher than in the controls [161/183 (88.0%) vs. 71/103 (68.9%), respectively, *p* = 0.001]. Additionally, a statistically significant difference was detected between active and inactive RR-MS patients [161/183 (88.0%) vs. 89/112 (79.5%), respectively, *p* = 0.048; Figure 1].

As EDSS 3 is considered as a clinical milestone separating patients with and without significant disability, we dichotomized MS patients into two groups according to the baseline EDSS as follows: ≤3.0 vs. ≥3.5. There was no statistically significant difference in IL-6 detectability in the total MS cohort between those two groups (*p* = 0.236). Additionally, a difference was not detected after the stratification of the whole group into two subgroups of relapsing (CIS and RR) and progressive (SP and PP) MS patients (*p* = 0.130 and *p* = 0.293, respectively).

The results of the logistic regression analysis of the predictive factors in RR-MS patients using disease activity as the dependent variable are presented in Table 3. Age, duration of MS, EDSS score, and CSF IL-6 detectability were predictive factors for disease activity as obtained by univariate analysis. In order to control potential confounding factors, we performed multivariate analysis in which age and EDSS at the time of LP were independent predictive factors for disease activity in our setting. Younger age and higher EDSS score at baseline were independent predictors of disease activity (Table 4).

**Table 4:** Logistic regression analysis of potential predictive factors for disease activity in patients with relapsing–remitting multiple sclerosis.

	Univariate analysis			Multivariate analysis		
Variable	OR	95% confidence interval	*P*	OR	95% confidence interval	*p*
Duration of MS	1.07	1.02–1.12	**0.006**			
Gender	1.16	0.71–1.92	0.552			
Age	1.06	1.03–1.08	**0.001**	1.06	1.04–1.08	**0.001**
EDSS score	0.79	0.64–0.97	**0.026**	0.72	0.56–0.91	**0.006**
OCB	0.59	0.34–1.04	0.068			
Previous number of relapses	0.90	0.67–1.20	0.473			
IL-6	1.89	1.08–3.58	**0.041**			

*Dependent variable: disease activity. Values in bold denote statistical significance. EDSS, expanded disability status scale; IL, interleukin; MS, multiple sclerosis; OCB, oligoclonal bands; RR-MS, relapsing–remitting MS*.

## Discussion

IL-6 is a classic cytokine featuring pleiotropic functions and that mediates a number of biological activities, including immune response regulation. It is involved in acute inflammatory response by inducing the synthesis of acute phase proteins (Heinrich et al., 1990). IL-6 also plays a key role in adaptive immune response. In particular, together with transforming growth factor β (TGF-β), it promotes the differentiation of naïve CD4^+^ T cells into T helper 17 cells and inhibits TGF-β-induced regulatory T cell (Treg) differentiation (Bettelli et al., 2006; Mangan et al., 2006; Veldhoen et al., 2006). The dysregulation of IL-6 could therefore alter the Th17/Treg balance, leading to autoimmune diseases (Kimura and Kishimoto, 2010). Accordingly, enhanced IL-6 signaling in serum and tissues has been found in rheumatoid arthritis, systemic lupus erythematosus, and RR-MS patients (Linker-Israeli et al., 1991; Maimone et al., 1997; Hirano, 1998). In MS, increased serum concentrations of IL-6 have been reported, particularly in patients with longer disease duration (Stelmasiak et al., 2000). Moreover, enhanced IL-6 release has been documented in monocytes from MS patients (Fiedler et al., 2017).

In the present study, conducted in a large cohort of MS patients with various disease phenotypes, we found that the presence of IL-6 in the CSF was observed more frequently in patients in comparison to a group of control subjects. Previous studies conducted in small samples of patients have reported increased CSF levels of various inflammatory molecules in MS patients compared with those in controls, although with inconsistent results (Kothur et al., 2016).

One study described higher CSF IL-6 detectability in patients with either MS or other inflammatory CNS diseases compared with patients with non-inflammatory neurological disorders (Maimone et al., 1991). However, other studies failed to demonstrate different CSF IL-6 levels between MS patients and controls (Miljkovic et al., 2002; Kothur et al., 2016; Matejčíková et al., 2017). In addition, higher CSF IL-6 concentrations have been found also in patients with other inflammatory conditions. In particular, increased CSF IL-6 levels have been reported in neuromyelitis optica in comparison to both RR-MS and other non-inflammatory neurological diseases (Matsushita et al., 2013; Kimura et al., 2017). Overall, these data suggest that increased CSF IL-6 concentrations could represent a nonspecific marker of CNS inflammation in line with the crucial role played by this molecule in the inflammatory processes (Göbel et al., 2018).

We found an increased detectability of IL-6 in CIS, RR, and SP-MS groups compared to that in controls. Interestingly, no significant difference in IL-6 detectability emerged between MS phenotypes. Preclinical and clinical studies have proposed that specific pathogenic mechanisms are involved in RR and progressive MS, including different immune profiles and cytokine expression (Lassmann et al., 2012). Our results suggest that IL-6 signaling is associated with MS regardless of disease phenotype, possibly indicating that IL-6 actions are involved in various pathogenetic mechanisms relevant to both RR and progressive MS.

An important finding of this study was the positive association between CSF IL-6 detectability and disease activity in patients with RR-MS. This result is in line with previous studies showing an increased expression of proinflammatory molecules in the CSF of MS patients during acute relapses, including IL-1β and CCL5/RANTES (Rossi et al., 2012; Mori et al., 2016). In particular, increased CSF levels of IL-6 have been previously reported in relapsing MS patients (Matsushita et al., 2013). Moreover, an enhanced expression of IL-6 has been found in both acute and chronic active demyelinating lesions (Maimone et al., 1997). It has been suggested that a proinflammatory CSF milieu is crucial in inducing and maintaining the inflammatory immune response in MS (Göbel et al., 2018). Accordingly, previous studies have shown that increased CSF levels of different proinflammatory molecules at the time of diagnosis promote a worse disease course. In particular, elevated IL-8 CSF levels in RR-MS patients have been associated to higher disease activity and increased risk of conversion to MS in patients with RIS and CIS (Rossi et al., 2015). Specifically, increased CSF IL-6 levels at the time of diagnosis have also been associated to a higher number of relapses and increased disability in RR-MS patients during a 3-year-long follow-up period (Stampanoni Bassi et al., 2018). Thus, these data confirm that IL-6 is a proinflammatory molecule significantly involved in acute CNS inflammation and, based on previous findings, also in MS prognosis.

Previous studies in small groups of MS patients have described correlations between serum and CSF IL-6 levels and EDSS score at the time of diagnosis (Stelmasiak et al., 2000; Kimura et al., 2017). In the present study, IL-6 detectability was not associated with EDSS at the time of diagnosis. Additionally, we found an association of IL-6 detectability neither with age nor with disease duration. Notably, it has been already demonstrated that aging might be associated with increased CNS inflammation and the enhanced expression of different proinflammatory molecules, including IL-6, playing a role in aging-related decline of neuronal function and synaptic plasticity impairment (Viviani and Boraso, 2011; Musella et al., 2018). In addition, we have previously reported that the IL-6 CSF concentration in RR-MS positively correlated with disease duration, suggesting that a prolonged time interval between disease onset and diagnosis could be associated to exacerbated CSF inflammation (Stampanoni Bassi et al., 2018). The present findings might therefore indicate that CSF IL-6 detectability does not change during disease progression since there is no significant difference in IL-6 detectability between MS phenotypes. However, a significantly increased detectability of IL-6 in CIS and RR-MS, in comparison to controls, speaks in favor of its presence in the CSF already in the early stages of MS.

Longitudinal studies are more robust for the assessment of predictive values of certain variables for reaching selected outcomes. However, we performed a cross-sectional analysis in order to estimate the predictive value of demographic and clinical/paraclinical variables without time component using logistic regression analysis. An additional potential limitation of this study might be that the difference in the age between patients and controls could be a potential source of confounding. Furthermore, in the present study, we only examined IL-6 CSF detectability. Therefore, the role of other proinflammatory and anti-inflammatory cytokines should be further investigated.

Overall, in line with previous studies, our present findings suggest that some CSF proinflammatory molecules could be useful for identifying those MS patients who are prone to increased disease activity. IL-6 could represent an interesting prognostic biomarker, although not specific, as increased CSF IL-6 concentrations have been linked to worse disease severity also in neurological inflammatory conditions other than MS (Matsushita et al., 2013; Wang et al., 2015).

## Data Availability Statement

The datasets generated for this study are available on request to the corresponding author.

## Ethics Statement

The studies involving human participants were reviewed and approved by Ethics Committees of the University Tor Vergata Hospital in Rome and of Neuromed Research Institute in Pozzilli, Italy. The patients/participants provided their written informed consent to participate in this study.

## Author Contributions

MS, LG, DC, and FB contributed in the conception and the design of the study. LG, GM, and FS organized the database. TP performed the statistical analysis. MS and EI wrote the first draft of the manuscript. JD, TP, RF, and AF wrote the sections of the manuscript. All authors contributed to manuscript revision, read, and approved the submitted version.

## Conflict of Interest

The authors declare the following potential conflicts of interest with respect to the research, authorship, and/or publication of this article RF has received honoraria as speaker or for research support from Biogen, Novartis, Merck, Roche, and Genzyme. GM received honoraria for speaking, consultation fees, and travel funding from Roche, Almirall, Bayer Schering, Biogen Idec, Merck Serono, Novartis, Sanofi-Genzyme, Mylan, and Teva. She is the principal investigator in clinical trials for Actelion, Biogen Idec, Merck Serono, Mitsubishi, Novartis, Roche, Sanofi-Genzyme, and Teva. FS acted as an Advisory Board member of Novartis, Biogen, and Merck Serono. DC is an Advisory Board member of Almirall, Bayer Schering, Biogen, GW Pharmaceuticals, Merck Serono, Novartis, Roche, Sanofi-Genzyme, and Teva and received honoraria for speaking or consultation fees from Almirall, Bayer Schering, Biogen, GW Pharmaceuticals, Merck Serono, Novartis, Roche, Sanofi-Genzyme, and Teva. He is also the principal investigator in clinical trials for Bayer Schering, Biogen, Merck Serono, Mitsubishi, Novartis, Roche, Sanofi-Genzyme, and Teva. His preclinical and clinical research was supported by grants from Bayer Schering, Biogen Idec, Celgene, Merck Serono, Novartis, Roche, Sanofi-Genzyme, and Teva. FB acted as an Advisory Board member of Teva and Roche and received honoraria for speaking or consultation fees from Merck Serono, Teva, Biogen Idec, Sanofi, and Novartis and non-financial support from Merck Serono, Teva, Biogen Idec, and Sanofi. The remaining authors declare that the research was conducted in the absence of any commercial or financial relationships that could be construed as a potential conflict of interest.
